# Unique double concentric ring organization of light harvesting complexes in *Gemmatimonas phototrophica*

**DOI:** 10.1371/journal.pbio.2003943

**Published:** 2017-12-18

**Authors:** Marko Dachev, David Bína, Roman Sobotka, Lenka Moravcová, Zdenko Gardian, David Kaftan, Václav Šlouf, Marcel Fuciman, Tomáš Polívka, Michal Koblížek

**Affiliations:** 1 Center Algatech, Institute of Microbiology of the Czech Academy of Sciences, Třeboň, Czech Republic; 2 Faculty of Science, University of South Bohemia, České Budějovice, Czech Republic; 3 Biology Center of the Czech Academy of Sciences, České Budějovice, Czech Republic; UMDNJ/Robert Wood Johnson Medical School, United States of America

## Abstract

The majority of life on Earth depends directly or indirectly on the sun as a source of energy. The initial step of photosynthesis is facilitated by light-harvesting complexes, which capture and transfer light energy into the reaction centers (RCs). Here, we analyzed the organization of photosynthetic (PS) complexes in the bacterium *G*. *phototrophica*, which so far is the only phototrophic representative of the bacterial phylum Gemmatimonadetes. The isolated complex has a molecular weight of about 800 ± 100 kDa, which is approximately 2 times larger than the core complex of *Rhodospirillum rubrum*. The complex contains 62.4 ± 4.7 bacteriochlorophyll (BChl) *a* molecules absorbing in 2 distinct infrared absorption bands with maxima at 816 and 868 nm. Using femtosecond transient absorption spectroscopy, we determined the energy transfer time between these spectral bands as 2 ps. Single particle analyses of the purified complexes showed that they were circular structures with an outer diameter of approximately 18 nm and a thickness of 7 nm. Based on the obtained, we propose that the light-harvesting complexes in *G*. *phototrophica* form 2 concentric rings surrounding the type 2 RC. The inner ring (corresponding to the B868 absorption band) is composed of 15 subunits and is analogous to the inner light-harvesting complex 1 (LH1) in purple bacteria. The outer ring is composed of 15 more distant BChl dimers with no or slow energy transfer between them, resulting in the B816 absorption band. This completely unique and elegant organization offers good structural stability, as well as high efficiency of light harvesting. Our results reveal that while the PS apparatus of Gemmatimonadetes was acquired via horizontal gene transfer from purple bacteria, it later evolved along its own pathway, devising a new arrangement of its light harvesting complexes.

## Author summary

The majority of life on Earth depends directly or indirectly on the sun as a source of energy. Phototrophic organisms use energy from light to power various cellular and metabolic processes. The initial step of photosynthesis is facilitated by light-harvesting complexes, which capture and transfer light energy into the reaction centers where it is used to power proton gradients or to form new chemical bonds. Here, we analyzed photosynthetic complexes in *Gemmatimonas phototrophica*, the only known phototrophic representative of the bacterial phylum Gemmatimonadetes. Using a combination of biochemical and spectroscopic techniques, we show that the light-harvesting complexes of *G*. *phototrophica* are organized in 2 concentric rings around the reaction center. This organization is unique among anoxygenic phototrophs. It offers both structural stability and high efficiency of light harvesting. The structural unit of both antenna rings is a dimer of photosynthetic pigments called bacteriochlorophyll. The inner ring is populated by more densely packed dimers, while the outer ring contains more distant dimers with a minimal excitation exchange. Such an arrangement modifies the spectral properties of bacteriochlorophylls in the complex and ensures efficient capture of light in the near-infrared part of the solar spectrum.

## Introduction

Photosynthetic (PS) microorganisms play an important role in many of Earth’s ecosystems due to their ability to harvest light and convert it to metabolic energy [1]. So far, phototrophic species were found in 7 bacterial phyla: Cyanobacteria, Proteobacteria, Chlorobi, Chloroflexi, Firmicutes, Acidobacteria, and Gemmatimonadetes [2]. The conversion of light into metabolic energy occurs in reaction centers (RCs) that carry out charge separation. Based on the terminal electron acceptor, the RCs can be divided in two groups [3]. Type 1 RCs, which use Fe-S clusters, are present in Chlorobi, Firmicutes, and Acidobacteria. Type 2 RCs, which use quinones, are possessed by Chloroflexi, Proteobacteria, and Gemmatimonadetes. Cyanobacteria are the only phototrophic prokaryotes that can evolve oxygen and possess both RC types.

The latest group found to contain phototrophic representatives is the phylum Gemmatimonadetes [4,5]. This phylum was formally established in 2003, with *G*. *aurantiaca* as a type species [6]. Its only cultured phototrophic representative is *G*. *phototrophica*, which was recently isolated from a freshwater lake in the Gobi Desert [7,8]. *G*. *phototrophica* contains bacteriochlorophyll (BChl) *a* as a main light-harvesting pigment and a large quantity of carotenoids. Its photosynthesis genes are organized in a 42.3-kb photosynthesis gene cluster (PGC) whose organization closely resembles that of Proteobacteria [7]. Also, phylogenetic analysis of the PS genes confirmed their homology to Proteobacteria. Based on these facts, it was suggested that phototrophy in Gemmatimonadetes originated from an ancient horizontal gene transfer event of a complete PGC from a purple PS bacterium [7]. If true, *G*. *phototrophica* represents the first known example of horizontal gene transfer of a complete set of photosynthesis genes between phototrophic and nonphototrophic representatives of distant bacterial phyla [2,7].

The environmental significance and distribution of phototrophic Gemmatimonadetes is not completely clear. These organisms are photoheterotrophic species, which require organic carbon for their metabolism and growth, but they can supplement a large part of their energy requirements using light-derived energy. Based on the analyses of available metagenomes, the highest proportion of phototrophic Gemmatimonadetes was found in wastewater treatment plants, soils, lake waters and sediments, estuarine waters, biofilms, plant-associated habitats, estuaries, and intertidal sediments. In contrast, no sequences from phototrophic Gemmatimonadetes were found in marine waters [9].

Little is known about the PS apparatus of *G*. *phototrophica*. The presence of the *puf* operon in its genome indicates the presence of type 2 RCs homologous to RCs of phototrophic Proteobacteria. The in vivo absorption spectrum of *G*. *phototrophica* reveals 2 main bands (819, 866 nm) in the near infrared region (NIR) [7]. This resembles the spectra of many phototrophic Proteobacteria that possess two types of light-harvesting complexes, which serve to both increase cross-section and expand spectral range of the RCs [10,11]. The inner antenna LH1 subunits encircle the RC, forming together the LH1-RC core complex [12–14]. The outer antenna light-harvesting complexes 2 (LH2) are organized in small rings placed in physical contact with the core complex. Interestingly, the genome of *G*. *phototrophica* does not contain any LH2 genes [7], so the identity of its 2 NIR absorption bands is unknown. The second characteristic of *G*. *phototrophica* is the presence of a large amount of carotenoids responsible for a strong absorption in the blue-green spectral region [7]. The light harvesting role of these pigments is uncertain since the heterotroph *G*. *aurantiaca* contains a similar set of carotenoids.

In order to elucidate the organization of the PS apparatus in *G*. *phototrophica*, we purified its PS complexes and performed a detailed biochemical and spectroscopic characterization.

## Results and discussion

### Characterization of the PS complexes from *G*. *phototrophica*

The released PS membranes from *G*. *phototrophica* contained 2 clearly visible absorption bands in the NIR and a large amount of carotenoids (S1 Fig). The PS complexes were purified by a combination of anion-exchange and size-exclusion chromatography (for details see Materials and methods). During chromatography, the majority of carotenoids eluted differently from the PS complexes (S2 Fig). This indicates that most of the carotenoids present in *G*. *phototrophica’s* membranes are not bound to PS complexes and do not serve for light harvesting.

Based on the retention time during the size-exclusion chromatography and native gel electrophoresis, we estimated that the *G*. *phototrophica* PS complex has a molecular weight of approx. 800 ± 100 kDa (S3A Fig), which is about 2 times larger than the LH1-RC core complex in *R*. *rubrum* (approximately 400 kDa). Further separation of *G*. *phototrophica* PS complex by sodium dodecyl sulfate (SDS)-electrophoresis have identified only 6 main protein bands in the range between 2 and 40 kDa. The 2 most intense bands, with apparent molecular masses approximately 5 kDa, most likely originated from light-harvesting antenna subunits (S3B Fig). The purified complex contained 62.4 ± 4.7 BChl *a* molecules (mean ± SD, *n* = 4). Both results showed that the PS complexes in *G*. *phototrophica* are much larger than the core complexes of *R*. *rubrum*.

The activity of the purified complex was verified by flash photolysis. The flash-induced difference spectrum was highly similar (S4 Fig) to the spectra previously recorded in purple PS bacteria [15], confirming that the RC of *G*. *phototrophica* is of the purple-bacterial type. The functionality was further confirmed using variable fluorescence measurements, which documented the high efficiency of primary charge separation (*F*_*V*_*/F*_*M*_ approximately 0.62) and active electron transfer (S5 Fig). Interestingly, the isolated complex retained fully functional photochemistry up to 60°C, which far exceeded *G*. *phototrophica’*s growth optimum of 25−30°C [8]. Such high thermal stability indicates a robust architecture of the studied PS complexes.

**Fig 1:**
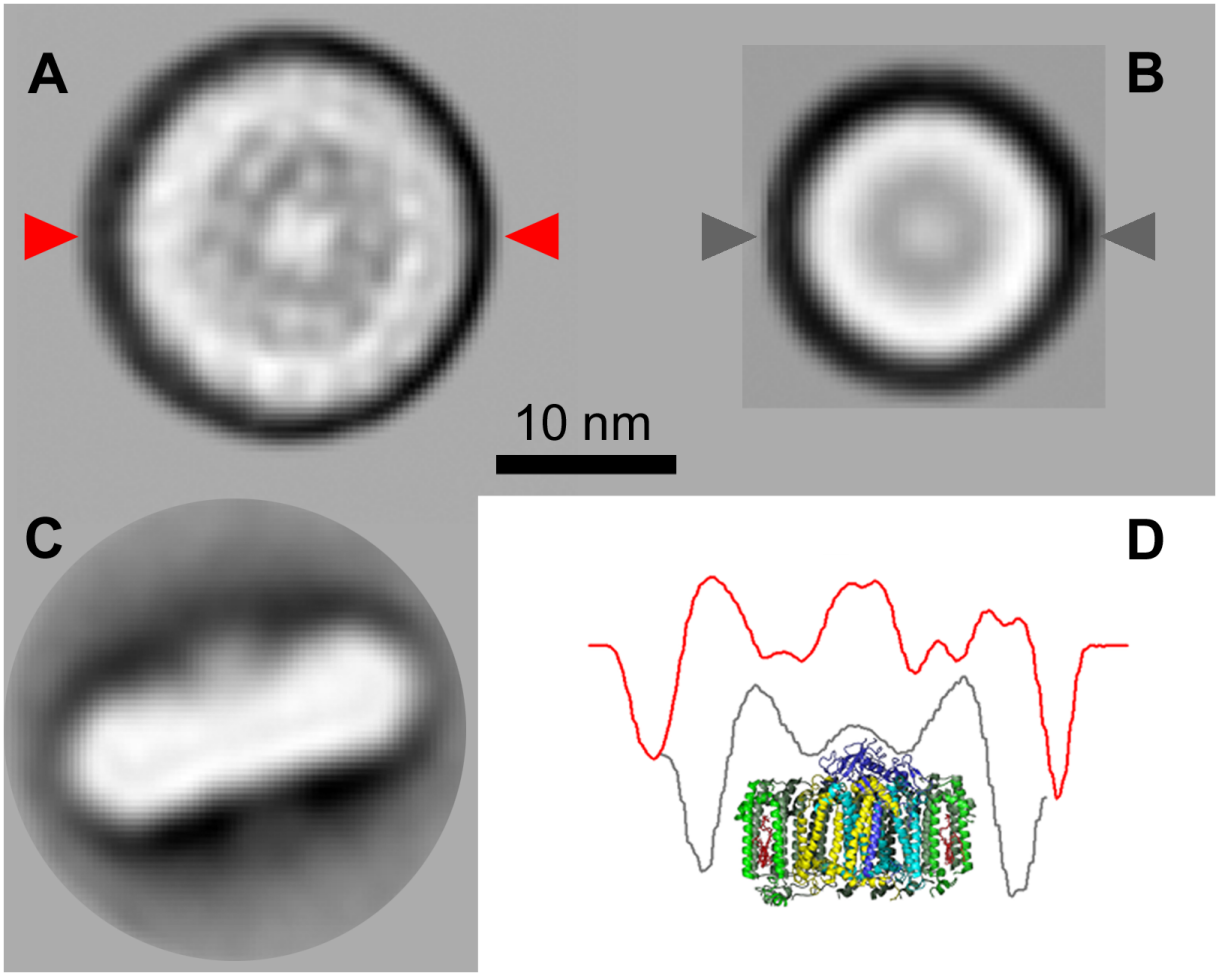
Top view projection maps of the PS complexes of *G*. *phototrophica* (A) and *R*. *rubrum* (B). Side view of an individual PS complex of *G*. *phototrophica* (C). Symmetric cross-sectional profile of the PS complexes of *G*. *phototrophica* (red line) and *R*. *rubrum* (grey line) super imposed on *R*. *rubrum* structural model (D). The position of the cross sections is indicated by small triangles on the projection maps A and B. PS, photosynthetic.

To obtain information about the overall structure of the light-harvesting systems of *G*. *phototrophica*, we analyzed the purified PS complexes using single particle analysis. The raw transmission electron microscopy (TEM) image revealed a large quantity of circular complexes (S6 Fig). The averaged image of the PS complex revealed a roughly circular structure with an apparent outer diameter of 198 Å (Fig 1). Assuming a 10 Å layer of detergent, one can estimate the net dimension of the PS complex to approximately 18 nm. The side-view projection showed elongated structures, frequently with a bulge on 1 side of the complex (thickness including the bulge of approximately 72 Å), probably representing the attached cytochrome (*pufC* gene product). For comparison, we also performed single particle analysis of the purified RC-LH1 complex of *R*. *rubrum*, which has an outer diameter of 13 nm. This means that the PS complex of *G*. *phototrophica* occupies an approximately 2 times larger membrane area when compared to the RC-LH1 complex of *R*. *rubrum*.

### Steady-state spectra

**Fig 2:**
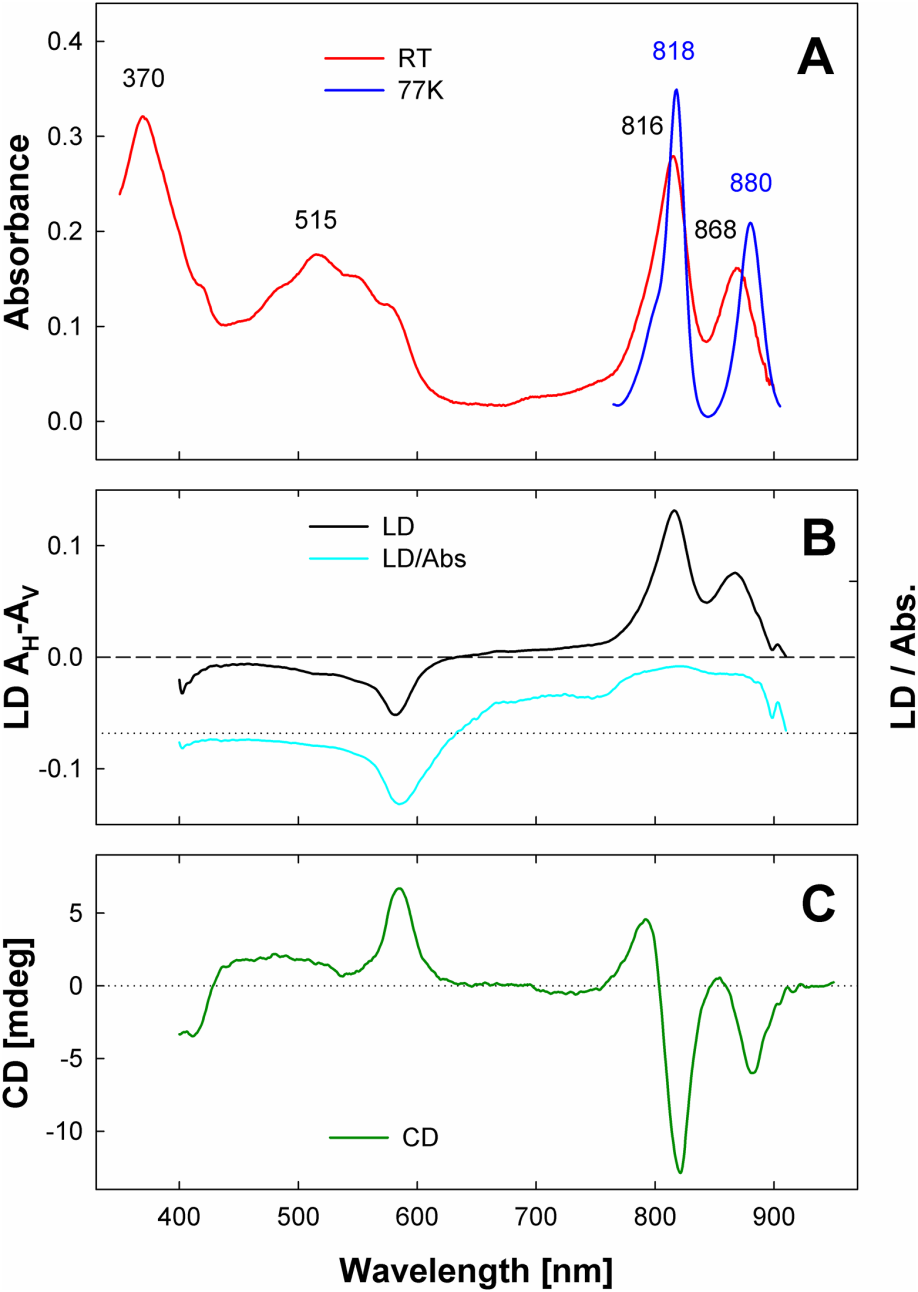
Steady-state spectra of purified PS complexes from *G*. *phototrophica*. (A) Absorption spectra recorded at room temperature (red line) and at 77 K (blue line). (B) The thick line shows the LD (*LD* = *A*_H_—*A*_V_) spectra of the PS complex embedded in polyacrylamide gel. *A*_H_ and *A*_V_ correspond to absorbance of horizontally and vertically polarized light, respectively. For a flat, disk-like particle in a vertically compressed gel, the horizontal direction is parallel with the particle plane, vertical with particle normal. The thin line shows the reduced LD, *LD* / *Abs*., where *Abs*. is isotropic absorbance. (C) Circular dichroism spectrum of PS complexes in solution. All dichroic spectra were measured at room temperature. Abs, absorbance; CD, circular dichroism; LD, linear dichroism; mdeg, millidegree; PS, photosynthetic; RT, room temperature.

The isolated complexes were further characterized by steady-state spectroscopy (Fig 2). The UV part of the spectrum was dominated by the Soret band of BChl *a* peaking at 370 nm (Fig 2A). Carotenoids cover an absorption range between 430–570 nm with the maximum at 515 nm. The vibrational sub-bands of the carotenoid spectrum were not well resolved. The minor absorption band at 575–595 nm seems to originate from the overlapping carotenoid 0–0 transition and the Q_x_ transition of BChl *a*. In the NIR region, the spectrum was characterized by BChl *a* bands peaking at 816 and 868 nm; in the following, these spectroscopic species will be denoted as B816 and B868, respectively. The ratio of amplitudes B816:B868 was approximately 1.7. Lowering of the temperature to 77 K led to the narrowing of both BChl *a* absorption bands and a shift of their maxima to longer wavelengths (Fig 2A). The shift was much more pronounced in the case of B868 (12 nm versus 2 nm of B816). Such a large decrease of the transition energy upon cooling is characteristic of the excitonically coupled pigment pools (B870 and B850) of LH1 and LH2 complexes [16]. At 77 K, the blue edge of the B816 resolved into a well-defined shoulder at approximately 800 nm.

Linear dichroism (LD) spectrum of the PS complex embedded in the vertically compressed gel is shown in Fig 2B. Assuming that under compression, the preferential orientation of the plane of the flat, disk-like complex was horizontal (normal vertical), the Q_y_ transitions of both the B816 and B868 were oriented predominantly parallel to the plane of the complex. The larger value of the LD/Absorbance ratio of B816 compared to B868 suggested that the Q_y_ dipole moments of the B816 BChls were slightly more in-plane than those of B868 in contrast to the B800 BChl *a* of LH2 and the B808 of B808-B866 from Chloroflexi [17–19]. The Q_x_ peak was observed at 583 nm and suggested that the corresponding dipole was oriented along the complex normal (vertically in the present geometry). The carotenoids exhibited very low LD.

The circular dichroism (CD) spectrum was dominated by BChl *a*. Carotenoids contributed only a minor broad positive band in the 430–550 nm region (Fig 2C) similar to CD of whole PS membranes of *R*. *rubrum* [20]. In contrast, CD spectra of isolated antenna complexes (LH1, LH2, B808-866) typically exhibit large, often nearly conservative carotenoid bands [19,21–23]. The BChl *a* contribution consisted of positive peaks at approximately 582 nm, 795 nm, and 855 nm, and negative bands peaking at 820 and 880 nm. Although the CD spectrum of the B868 region could be easily interpreted as a LH1-like BChl *a* aggregate, the B816 region deserves more attention. The large asymmetry between the positive and negative lobe of the CD spectrum, accompanied with a large, 13 nm blue-shift of the zero-crossing point with respect to the maximum of the absorption band are not typical of LH2 or B808-866 complexes [19,21,22]. However, both features are present in the CD spectrum of the structural unit of the LH1 complex, B820, an excitonically coupled dimer of BChl *a* bound to α and β helices [20,24].

### Femtosecond transient absorption spectroscopy

**Fig 3:**
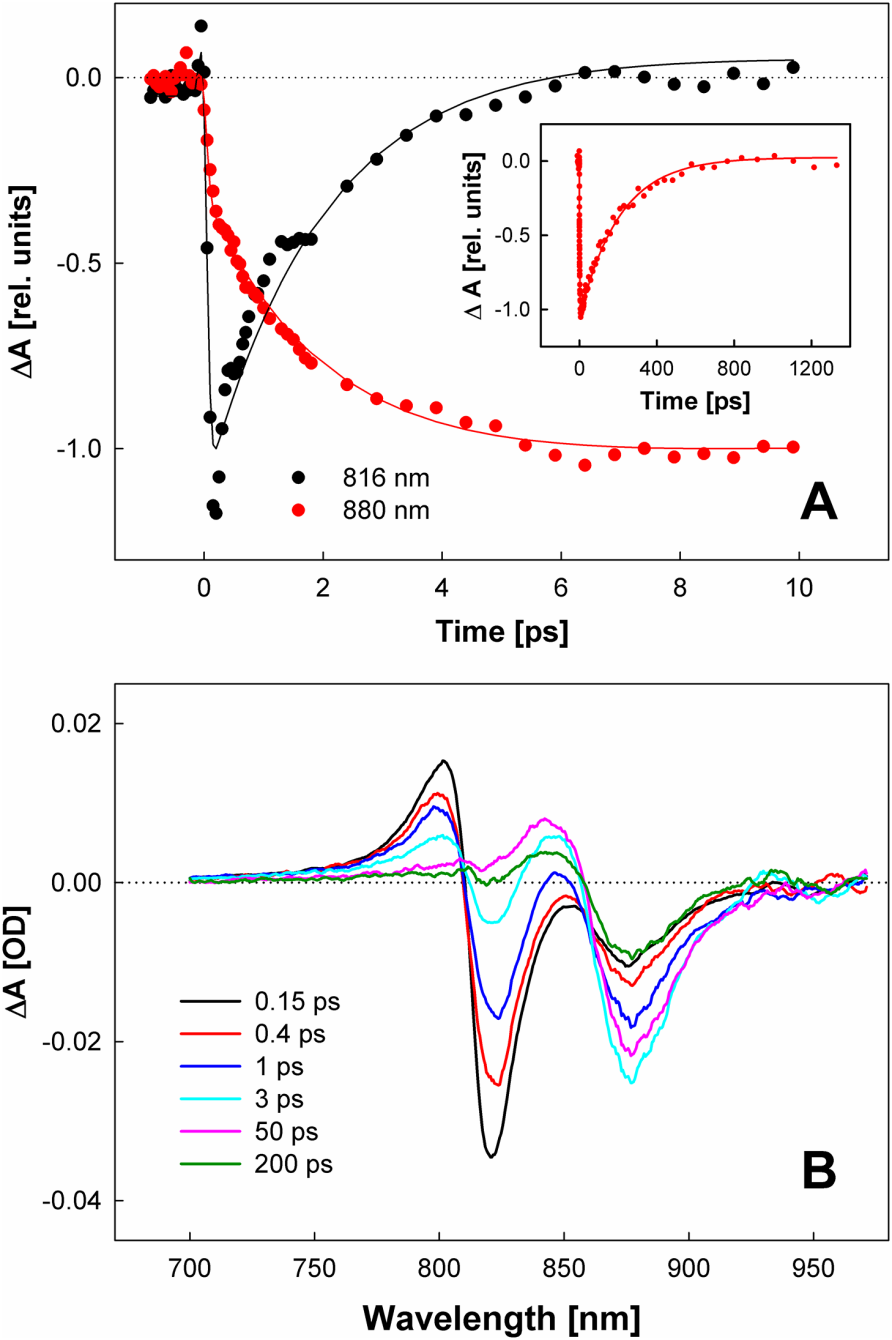
(A) Kinetics measured at 816 and at 880 nm after excitation of the complex at 820 nm. Kinetics are normalized to the bleaching maximum. Lines represent the fits obtained from global fitting. The inset shows the 880 nm kinetics over a longer timescale. (B) Representative transient absorption spectra recorded after excitation at 820 nm. OD, optical density; rel., relative.

To explore energy transfer between the B816 and B868 nm bands, we excited the complex at 820 nm and recorded transient absorption spectra in the 700–970 nm spectral window. Fig 3A shows kinetics at the wavelengths corresponding to the ground-state bleaching of the both bands. The kinetics clearly demonstrate the energy transfer process: as the signal at 820 nm decays, the signal at 880 nm (red-shifted with respect to steady-state absorption because of the contribution from the stimulated emission and overlap with excited-state absorption) appears. Fig 3B shows the complementary transient absorption spectra, which provide information about the spectral evolution of the system.

Global fitting of the whole spectro-temporal dataset revealed time constants of 2 ps and 210 ps (S6 Fig). The first time constant obviously characterizes the energy transfer between the B816 and B868 bands because it is associated with the decay of the B816 band and concomitant rise of the B868 band (Fig 3A and S7 Fig). The B816-B868 energy transfer time of 2 ps is slightly longer but comparable to the B800-B850 energy transfer in LH2 complexes: *Rhodobacter sphaeroides* (0.7 ps) [25], *Rhodopseudomonas acidophila* (0.8–0.9 ps) [26,27], *Thermochromatium tepidum* (0.8–0.9) [28], *Rhodospirillum molischianum* (1.0 ps) [29]. A similar situation was found in Chloroflexi, which contain type 2 RCs surrounded by a circular antenna, which in this case binds 2 different pools of pigments [19,21]. Here, the energy transfer times in the core complex of *Chloroflexus aurantiacus* [30] and *Roseiflexus castenholzii* [31] were almost the same as in *G*. *phototrophica*. The slower kinetics, observed in the B868 band, populated by energy transfer from B816, have a lifetime of 210 ps (S7 Fig), which thus characterizes B868-RC energy transfer (Fig 3A, inset).

The shape of transient absorption spectra can also provide some information about arrangement of BChl *a* molecules within the PS complex of *G*. *phototropica*. The ground-state bleaching signals of both B816 and B868 bands are accompanied by positive, blue-shifted excited-state absorption bands. This pattern is well-known from systems containing excitonically coupled BChls, such as LH1 [32] or LH2 [33] complexes of purple bacteria. The *G*. *phototrophica* light-harvesting complex is thus likely a system in which both B816 and B868 bands exhibit signatures of excitonic coupling. It is also worth mentioning that the zero-crossing point in the transient absorption spectrum at approximately 810 nm hardly moved with time (Fig 3B). Essentially the same behavior was recorded for the B820 complex, whereas in the LH1 complex the zero-crossing point shifted over time due to equilibration among LH1 subunits [32]. Thus, as for the CD spectra described above, the dynamic behavior of the transient absorption spectra also points to the B816 band as being composed of BChl *a* dimers with no or slow energy transfer between them.

### Working model of the *G*. *phototrophica*’s PS complex

All the collected structural and spectroscopic data provide evidence for some unique features of *G*. *phototrophica*’s PS complex. It is an approximately circular aggregate with an outer diameter of approximately 18 nm. The complex contains 62.4 ± 4.7 BChl *a* molecules per RC. This number is almost identical to the value determined previously from the whole cell extracts [7], which indicates that the number of BChl *a* molecules in the complex is fixed and is not dependent on growth conditions. This number also far exceeds the pigment pools of 30–36 BChl *a* molecules per RC observed in LH1–RC complexes of Proteobacteria [14,34]. These considerations led to a double concentric ring organization of the *G*. *phototrophica* PS complex with a densely packed inner part, similar in dimension to LH1 for B868 and a loosely spaced outer shell of B816. To determine the number of subunits, we analyzed the angular distances of the subunits observed on the peripheral part of the complex. The mean angular distance of the apparent subunits was 24 degrees, which corresponds to the 15-meric symmetry (S8 Fig). We assume that the BChl *a* molecules are divided into 3 pools—4 molecules as a part of the RC, 30 molecules forming an inner (LH1-like) ring around the RC, and 30 molecules forming the second peripheral ring (for details see the discussion in S1 Text). The structural unit of both pigment pools can be assumed to be a 2-helix–2BChl *a* complex. This predicted organization with 2 concentric rings composed of 15 dimers each harboring 2 BChl *a* molecules translates in total to 60 BChl *a* molecules, which is consistent with the number of BChl *a* determined by the liquid chromatography.

**Fig 4:**
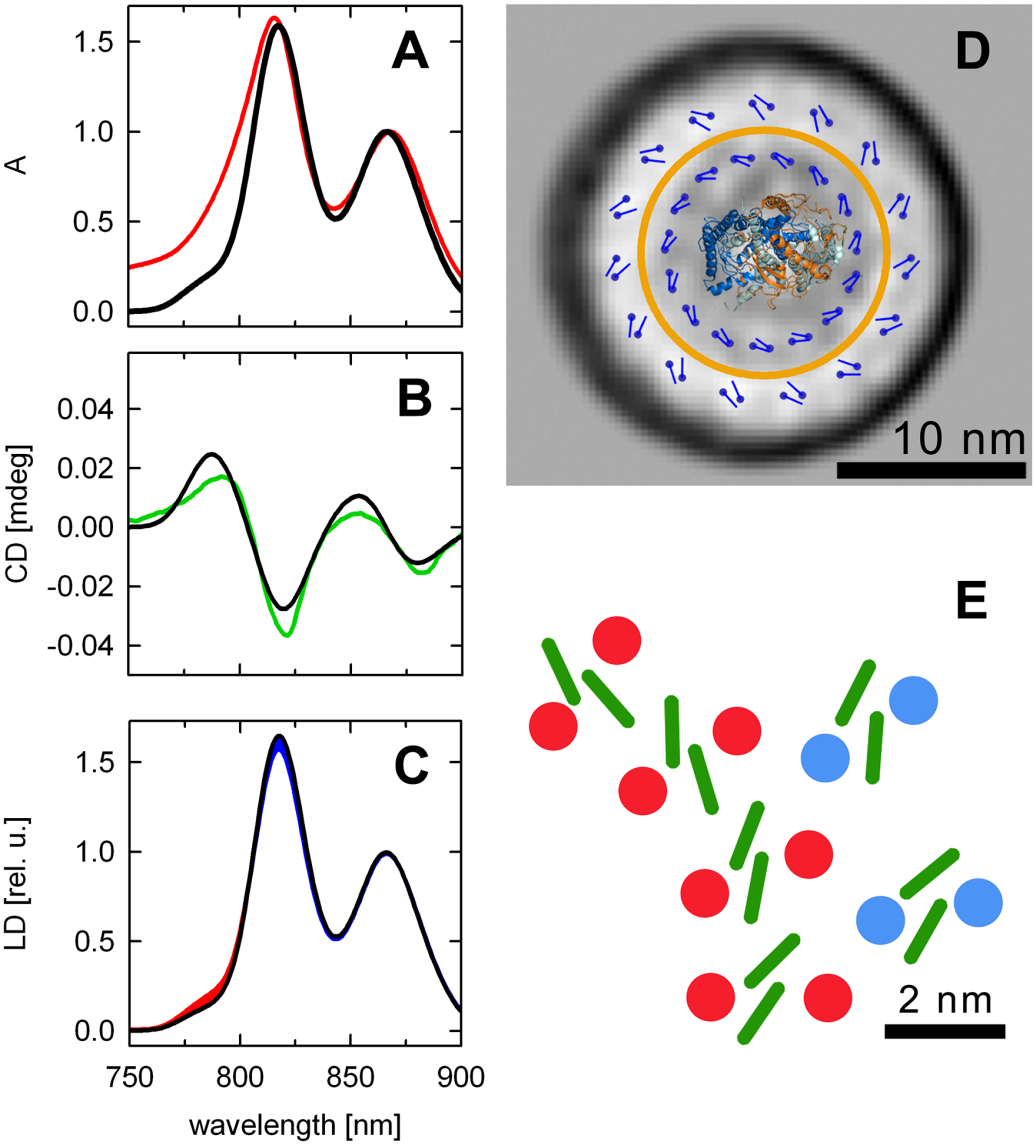
Model of *G*. *phototrophica* light-harvesting complex. Panels on the left show (A) absorption, (B) CD, and (C) LD spectra. The recorded NIR spectra (in color) are compared with the simulated steady-state spectra (in black). Blue area in (C) depicts the spectral region having LD > absorbance, red area marks the region with LD < absorbance (after normalization to B868 maximum). (D) Arrangement of BChl molecules in the complex used to calculate the steady-state spectra in (A-C). Yellow ring indicates the size of the LH1 complex from *R*. *rubrum* for comparison. (E) Detail of the section of the complex showing the hypothetical position of protein helices (red and blue dots) and BChl *a* molecules in green. These are taken directly from the structure of light-harvesting complexes. BChl, bacteriochlorophyll; CD, circular dichroism; LD, linear dichroism; LH1, light-harvesting complex 1; mdeg, millidegree; NIR, near infrared region; rel. u., relative units.

To verify our theoretical prediction, we calculated the steady-state NIR spectra (absorbance, CD, LD) using a point-dipole approximation [35] for such pigment geometry and compared the simulated spectra with the measured ones (Fig 4A–4C). The simulation started from a 2-helix–2BChl *a* building block (Protein Databank Identifier:2FKW) repeated so as to produce the required 2 rings with 15-meric symmetry, in a similar experiment to that done by Georgakopoulou et al. [23]. Parameters used in the cited work to simulate the LH1 spectra were used as a starting point. The dipole directions and transition energies were then adjusted to match the measured spectra of *G*. *phototrophica* PS complex, first manually and then fine-tuned using a genetic algorithm. The full set of parameters used to compute the steady-state spectra is presented in S1 Table.

As seen in Fig 4A–4C, the majority of the features of the experimental steady-state spectra are quantitatively accounted for by the given parameters, including the blue-shift of the B816 crossing point (Fig 4B), the slightly higher orientation of the B816 dipoles compared to B868 (Fig 4C), and a decrease of polarization in the blue edge of the B816 band (Fig 4C), due to the overlap of several excitonic components. On the other hand, the model failed to predict the extent of the nonconservative nature of the CD signals. However, this was expected because it was shown before (e.g., ref. [23]) that the inclusion of the interaction of BChl *a* Q_y_ with higher energy transitions, such as Soret bands, Q_x_ and carotenoids is necessary to produce the required degree of asymmetry in the CD bands. The relative difference of the intensity of the B816 and B868 bands is accounted for by less than 20% of the relative increase of the transition dipole moment of BChl *a* bound to B816 compared to B868, which is well within the values used to simulate spectroscopic properties of LH1 [23].

The above considerations led us to propose the model shown in Fig 4D. As expected, the dominant pigment–pigment interactions were found within the BChl *a* dimers (289 cm^−1^ and 220 cm^−1^ for B868 and B816 subunits, respectively). The strongest computed interaction between neighboring dimers was 55 cm^−1^ in the B868 ring. This is more than 5 times lower compared to typical inter-dimer interactions of both LH1 or LH2 complexes. This can be partially accounted for by the fact that the simulation was performed for a 15-meric ring with a diameter corresponding to the standard 16-meric LH1, leading to the larger separation between closest BChls of neighboring dimers, but it also likely indicates a difference in the detailed geometry of the pigments. The strongest inter-dimer interaction within the B816 ring was less than 7 cm^−1^ due to the large distance between the dimeric subunits; hence, B816 consists effectively of isolated dimers. The strongest predicted coupling between B868 and B816 pigments was −12 cm^−1^. This is lower but comparable to the theoretically predicted B800–B850 couplings in LH2 [36,37] and in agreement with the observed excitation transfer times. In addition, because the present model of the *G*. *phototrophica* PS complex assumes concentric arrangement of dimeric subunits with the B868 forming an (approximately) LH1-like core surrounded by an external B816 antenna, it is of interest to compare it also to the functioning of the PS unit of LH2-containing purple bacteria. Here, the fastest LH2-LH1 transfer times were found in the range 3–5 ps [38,39] for the theoretically predicted electronic coupling between donor and acceptor states in the range of approximately 2–10 cm^−1^ [40].

For completeness, in Fig 4E we suggest the organization of the protein helices corresponding to the above described pigment geometry.

### Conclusions

Light-harvesting complexes in *G*. *phototrophica* harbor approximately 60 BChl *a* molecules arranged in 2 concentric rings surrounding the type 2 RC. This unique and elegant organization offers high efficiency of light absorption and excitation transfer as well as high structural stability. Our results also demonstrate that while the PS apparatus of Gemmatimonadetes was likely acquired via horizontal gene transfer from purple bacteria, it later evolved along its own trajectory devising a novel organization for its light-harvesting complexes.

## Materials and methods

### Cultivation

*G*. *phototrophica* strain AP64^T^ was grown on modified R_2_A agar media in a Memmert INCO 108med incubator under 90% N_2_, 10% O_2_ atmosphere at 28 ± 1°C, in the dark [8]. The medium was supplemented with 50 mg L^−1^ ampicillin to avoid bacterial contamination. The purity of the colonies was routinely checked using a custom made NIR microspectrometry system assembled from a Nikon SMZ800N stereomicroscope and a QE Pro-FL CCD spectrometer (Ocean Optics Inc., Largo, FL) connected via fiber optics. The colonies were harvested approximately 1 month after inoculation, using a plastic scraper and stored at −20°C until needed. *R*. *rubrum* was grown in cap-closed bottles on complex medium [41] on an orbital shaker.

### Purification of PS complexes

The harvested cells (collected from approximately 20 agar plates) were resuspended in buffer A (50 mM Tris, pH 8, 1 mM EDTA, 50 mM NaCl) and centrifuged for 10 min at 10,000 × g. The cells were broken using an EmulsiFlex-C5 (Avestin Inc., Ottawa, Ontario, Canada) at 140 MPa, and unbroken cells with cell debris were removed by centrifugation for 10 min at 5,000 × g. The released membranes were pelleted by ultracentrifugation (60 min, 110,000 × g) and resuspended in 0.5 mL buffer A containing 1 mM phenylmethylsulfonyl fluoride. Subsequently, the membranes were solubilized with a mixture containing 2% of n-dodecyl β-D-maltoside (β-DDM) and 0.2% of Triton-X100 at room temperature in the dark for 30 min. The separation of solubilized membranes was carried out using a Pharmacia FPLC system equipped with a UnoQ-6 ion-exchange column (Bio-Rad, Hercules, CA). The sample was loaded on top of the column and eluted in 20 mM HEPES, pH 8.0, 0.06% β-DDM, with linearly increasing concentration (from 0 to 0.5 M) of MgCl_2_ at a flow rate of 1 mL min^−1^ over 60 min. The signal was detected using a Prominence SPD-20AV 2-wavelength UV/VIS detector (Shimadzu Inc., Kyoto, Japan). The fractions containing PS complexes were pooled and concentrated on 100-kD cutoff micro-concentrators (Sartorius, Göttingen, Germany). The solubilized complexes were further purified by gel filtration using a Yarra SEC-3000 column (Phenomenex, Torrance, CA) and 20 mM HEPES, pH 8.0, with 0.2% β-DDM at a flow rate of 0.2 mL min^−1^ at 10°C. The gel filtration was performed using an Agilent 1200 system equipped with a UV-VIS-NIR diode-array detector and fraction collector. The collected pigment–protein complexes were kept on ice in the dark to prevent sample degradation.

### Electron microscopy and single particle analysis

Electron microscopy was performed on freshly prepared complexes (same day of purification). Samples were deposited on glow-discharged carbon-coated copper grids and negatively stained with 1.5% uranyl acetate, and visualized using a JEOL JEM–2100F transmission electron microscope (JEOL, Tokyo, Japan; using 200 kV at 20,000 × magnification). TEM images were recorded using a bottom-mounted Gatan CCD Orius SC1000 camera, with a resolution corresponding to 3.4 Å per pixel. Image analysis was carried out using RELION [42]. The selected projections were rotationally and translationally aligned, and treated by empirical Bayesian approach in combination with classification procedure to refine 2D class averages.

### Steady state absorption and fluorescence spectroscopy

Room temperature steady-state absorption spectra were recorded using a UV-VIS-NIR spectrometer UV2600 (Shimadzu) equipped with an integrating sphere. Low-temperature absorption was measured using an OptistatDN2 nitrogen cryostat. CD spectra at room temperature were recorded using a Jasco J-715 spectropolarimeter. LD spectra were recorded on samples embedded in 10% acrylamide gel [17]; cylinders of gel, 0.9 cm in diameter were vertically compressed to 60% of their original height in 1 × 1 cm cuvettes, leading to horizontal expansion of the gel.

### Femtosecond time-resolved absorption spectroscopy

The femtosecond time-resolved spectroscopy was conducted using a modular laser system assembled from a Spitfire Ace-100F ultrafast Ti-sapphire regenerative amplifier (Spectra-Physics, Santa Clara, CA) seeded with the Mai Tai SP oscillator (Spectra-Physics) and pumped with an Empower 30 laser (Spectra-Physics). The system produced pulses with a central wavelength of 800 nm, approximately 120 fs duration and a 1 kHz repetition rate. Part of the output power was used to prepare excitation (pump) pulses, another part to produce broad-band probe pulses. A gradually increasing delay between the 2 pulses was set by a computer-controlled delay line in the probe pathway. The desired excitation wavelength was tuned by means of an optical parametric amplifier (TOPAS; Light Conversion, Vilnius, Lithuania). The generation of supercontinuum for the probe pulses was achieved in a 2-mm sapphire plate by applying 1,100-nm pulses derived from another TOPAS. The mutual polarization between pump and probe was set to the magic angle (54.7°). The probe beam was split into 2: one served as a reference, the other overlapped spatially with the pump beam at the sample. Both broadband pulses were then directed into the spectrograph, in which they were dispersed onto a double CCD array. Prior to the measurements, the sample was diluted in a buffer to reach an optical density of approximately 0.4 at 820 nm in a 2-mm path length quartz cuvette. A microstirrer was used to continuously mix the sample during the measurements. The intensity of the pump pulses was kept below 10^13^ photons pulse^−1^ cm^−1^. The data were fitted globally using DAFit software (Pascher Instruments, Lund, Sweden), which employs a sequential kinetic scheme with increasing lifetimes.

### Other methods

Flash-induced absorbance spectra of purified PS complexes of *G*. *phototrophica* were measured using a laboratory-built kinetic spectrometer [43]. The spectrum was calculated as a light minus dark difference of absorption spectra recorded at 3 μs after xenon flash.

The pigment-protein complexes were analyzed by CN electrophoresis. For native electrophoresis, the membranes from *G*. *phototrophica* or *R*. *rubrum* were concentrated (Vivaspin 100K MW cut-off) and resuspended in buffer B containing: 25 mM MES/NaOH, pH 6.5, 10 mM MgCl_2_, 10 mM CaCl_2_, 25% glycerol. The buffer B was supplemented with 10% (DDM) in H_2_O [w/v]. The sample was mixed and spun down (18,000 × g, 10 min, 4°C) and subsequently loaded on 4%–14% clear native gel [44]. Colored bands corresponding to PS complexes of *G*. *phototrophica* and *R*. *rubrum* were cut from the native gel, incubated for 30 min in 2% SDS and placed on the top of the 12%–20% gradient SDS gel [45]. Separated proteins were visualized by Coomassie blue staining. Pigments were analyzed using a Prominence-i HPLC system (Shimadzu Inc.) equipped with a Phenomenex Luna 3μC8(2) 100 Å column using an ammonium acetate:methanol solvent system as described before [34]. The number of BChl *a* molecules per RC (PS unit size) was determined from the ratio of molar concentrations of BChl *a* and bacteriophaeophytin *a* multiplied by 2 (for details see ref. [34]).

## Supporting information

S1 Text Detailed discussion of the BChl *a* stoichiometry in PS complex. BChl, bacteriochlorophyll; PS, photosynthetic.
(DOC)

S1 Table Parameters used for computation of the steady-state optical spectra of *G*. *phototrophica* PS complex. PS, photosynthetic.
(PDF)

S1 Fig Absorption spectra of PS membranes from *G*. *phototrophica* (red) and *R*. *rubrum* (grey). PS, photosynthetic.
(TIF)

S2 Fig Upper panel: size exclusion chromatography of the partially purified PS complexes from *G*. *phototrophica*. The blue line recorded at 820 nm shows the fraction with the purified complex, and the red trace at 490 nm represent the carotenoids. Lower panel: the absorption spectrum of the obtained complex (blue) and the spectrum of the “free” carotenoids taken by the online diode array detector. PS, photosynthetic.
(TIF)

S3 Fig PS complex of *G*. *phototrophica* analyzed by native and SDS polyacrylamide gel electrophoresis. (A) Clear-native gel electrophoresis of *G*. *phototrophica* membrane complexes in comparison with membrane complexes of *R*. *rubrum* and *Synechocystis* sp. PCC 6803; the membranes were solubilized using 2% dodecyl-β-maltoside and loaded on 4%–14% clear-native gel (41). Abbreviations used: PSI[1] and PSI[3], monomer and trimer of PSI, respectively; PSII[2], dimer of PSII; CpcA/B[6], approximately 100 kDa heterohexamer of CpcA and CpcB phycobilinoproteins. (B) Colored bands corresponding to PS complexes of *G*. *phototrophica* and *R*. *rubrum* were cut from the native gel as indicated by dashed boxes in (A), incubated for 30 min in 2% SDS, and the proteins were separated by the gel electrophoresis. LH1 subunits of *R*. *rubrum* (α,β) are indicated by arrows. CpcA andCpcB, phycocyanine alpha and beta proteins; kDa, kiloDalton; PS, photosynthetic; SDS, sodium dodecyl sulfate.
(TIF)

S4 Fig Flash-induced absorbance spectra of purified photosynthetic complexes of *G*. *phototrophica*. As expected for the type 2 RC, the signal is dominated by signatures of the oxidized primary donor (P870^+^): bleaching around 865 nm, electrochomic shift of the accessory BChl *a* around 800 nm and bleaching of the Q_x_ band of the primary donor at 600 nm. BChl, bacteriochlorophyll; RC, reaction center.
(TIF)

S5 Fig BChl *a* fluorescence induction and relaxation recorded using the kinetic fluorometer FL-3000 (Photon Systems Instruments Ltd., Brno, Czech Republic). The BChl fluorescence induction transient was elicited by a 140 μs-long square-wave pulse of light with an intensity of approximately 0.1 mol *photon* m^−2^s^−1^ provided by an array of blue-green 505 nm Luxeon Rebel diodes. The signal was registered using a silicon photodiode detector (λ > 850 nm) with 10 MHz resolution. Inserts shows the *F*_V_*/F*_M_ values (*F*_V_*/F*_M_ = [*F*_M_-*F*_0_]*/F*_M_) recorded at temperature range 24–60°C revealing great temperature stability of the complex. BChl, bacteriochlorophyll.
(TIF)

S6 Fig Example of a raw TEM image of *G*. *phototrophica* complexes. TEM, transmission electron microscopy.
(TIF)

S7 Fig The spectro-temporal datasets obtained from the measurements were analyzed globally by fitting package DAFit (Pascher Instruments). To visualize the excited state dynamics, we assumed that the excited states evolved according to a sequential, irreversible scheme A → B, B → C, C → D. The arrows represent increasingly slower processes and the time constants of these processes correspond to lifetimes of the species A, B, C, D. The spectral profiles of the species are called EADS, and although in complex systems they do not directly correspond to the individual excited state species, they provide information about the time evolution of the whole system. This figure shows EADS obtained from global fitting of the data recorded after excitation of the complex at 820 nm. EADS, evolution-associated difference spectra.
(TIF)

S8 Fig Estimated angular distance between neighboring subunits of the PS complex. The angle was measured as shown in the insert. The data were derived from 23 measurements from 4 individual complex images. PS, photosynthetic.
(TIF)

S9 Fig Comparison of B816 and B868 absorption bands. Red line: absorption spectrum of *G*. *phototrophica* PS complex. Grey area: absorption spectrum of *R*. *rubrum* complex scaled and blue-shifted to match the B868 band. Blue area: the difference between the red and grey spectra, corresponds to the pure spectrum of the B816 absorption band. The ratio of B816 and B868 absorption band areas (blue to grey) was 1.35:1.0. PS, photosynthetic.
(TIF)

## Abbreviations

BChl: bacteriochlorophyll
CD: circular dichroism
LD: linear dichroism
LH1: light-harvesting complex 1
LH2: light-harvesting complex 2
NIR: near infrared region
PGC: photosynthesis gene cluster
PS: photosynthetic
RC: reaction center
SDS: sodium dodecyl sulfate
TEM: transmission electron microscopy
β-DDM: n-dodecyl β-D-maltoside
