# Supplementary material for: Unique double concentric ring organization of light harvesting complexes in Gemmatimonas phototrophica
Source: PLoS Biol. 2017 Dec 18;15(12):e2003943. doi: 10.1371/journal.pbio.2003943 (PMC5749889; doi:10.1371/journal.pbio.2003943)
Supplement: S1 Text — BChl, bacteriochlorophyll; PS, photosynthetic. (DOC) [file pbio.2003943.s001.doc]

**S1 Text**

*Discussion of the BChl a stoichiometry*

In the present study we determined that the PS complex of *G. phototrophica* binds 62.4 ± 4.7 BChl *a* molecules per reaction center. This value is almost identical to the number determined previously *in vivo* [1] and it seems to be constant under all tested growth conditions. This indicates that *G. phototrophica* does not regulate antenna size, and thus the organization is also likely unchanged. Based on phylogenetic analyses [1] the PS complex contains a standard purple-bacterial RC, which contains 4 BChl *a* + 2 BPheo *a* molecules. Thus, the LH complex consists of about 58 BChl *a* molecules in two spectral pools. The spectroscopic properties of the low-energy BChl *a* pool, B868, are consistent with the LH1-like BChl *a* aggregate. A circular aggregate can thus be assumed. The nature of the high-energy BChl *a* pool, B816, is not completely clear, however, both the steady-state and time-resolved data indicate a certain degree of excitonic character, compatible with a BChl *a* dimer, based on the similarities to the B820 subunit of the LH1 complex.

To estimate the pigment allocation in the respective antenna pools, we compared the areas of the B868 and B816 spectra (S9 Fig). Instead of using deconvolution into Gaussian bands, the area under B868 was estimated by fitting it with the absorption spectrum of the *R. rubrum* core. The B816 : B868 area ratio obtained was ~1.35 : 1. Assuming the same oscillator strength of the B816 and B868 BChl *a* this would suggest that the high-energy pool contains about 30% more pigment. However, since such variation in the BChl *a* dipole moment is within the range of what can be expected from environmental effects [2], we will for simplicity assume that B816 and B868 contain the same number of pigments. Considering that pigment spacing in the strongly excitonically coupled LH1 complexes is about 1 nm, ~60 pigments placed 1 nm apart can form a ring of about 19 nm in diameter, corresponding to the dimensions of the entire PS complex, leaving no pigments or space for the B816 pool. Consequently, the size of the B868 ring must be much smaller. Dividing the total of ~60 pigment molecules in half yields 30 BChl *a* per in B868 pool, which dictates a ~9.5 nm ring, which is comparable to the diameter of a regular LH1 complex. Moreover, it can be expected that the high-energy pigments are positioned in the outer parts of the complex to achieve excitation energy transfer towards the RC, absorbing at about 870 nm. The other 30 molecules are thus determined to be part of the B816 pool, which makes the total of 64 BChl *a* molecules per RC.

**References**

1. Zeng Y, Feng FY, Medová H, Dean J, Koblížek M. Functional type 2 photosynthetic reaction centers found in the rare bacterial phylum Gemmatimonadates. Proc Natl Acad Sci USA. 2014; 111:7795-7800.
2. Georgakopoulou S, Zwan GR, Olsen JO, Hunter CN, Niederman RA, van Grondelle R. Investigation of the effects of different carotenoids on the absorption and CD signals of light harvesting 1 complexes*.* J Phys Chem B. 2006; 110:3354-3361.
