# Supplementary material for: Unique double concentric ring organization of light harvesting complexes in Gemmatimonas phototrophica
Source: PLoS Biol. 2017 Dec 18;15(12):e2003943. doi: 10.1371/journal.pbio.2003943 (PMC5749889; doi:10.1371/journal.pbio.2003943)
Supplement: S1 Table — PS, photosynthetic. (PDF) [file pbio.2003943.s002.pdf]

S1 Table

Parameters used for computation of the steady-state optical spectra of *G. phototrophica* PS complex

	B868	B816
**μ (Debye)**	6.2	7.3
**E (cm^{-1}; nm)**	11995; 834	12478; 802
**ΔE (cm^{-1})**	278	12
**ϵ**	1.2	
**σ (cm^{-1})**	600	400
**Δ (cm^{-1})**	270	

μ: transition dipole moment; E: site energy; ΔE: energy difference between α and β BChls, i.e. within the dimeric structural unit of the antenna rings; ϵ: dielectric constant; σ: inhomogeneous broadening, FWHM of a Gaussian distribution for diagonal disorder of the B868 and B816; Δ: homogeneous broadening.
