# Supplementary figures and images for: Unique double concentric ring organization of light harvesting complexes in Gemmatimonas phototrophica
Source: PLoS Biol. 2017 Dec 18;15(12):e2003943. doi: 10.1371/journal.pbio.2003943 (PMC5749889; doi:10.1371/journal.pbio.2003943)

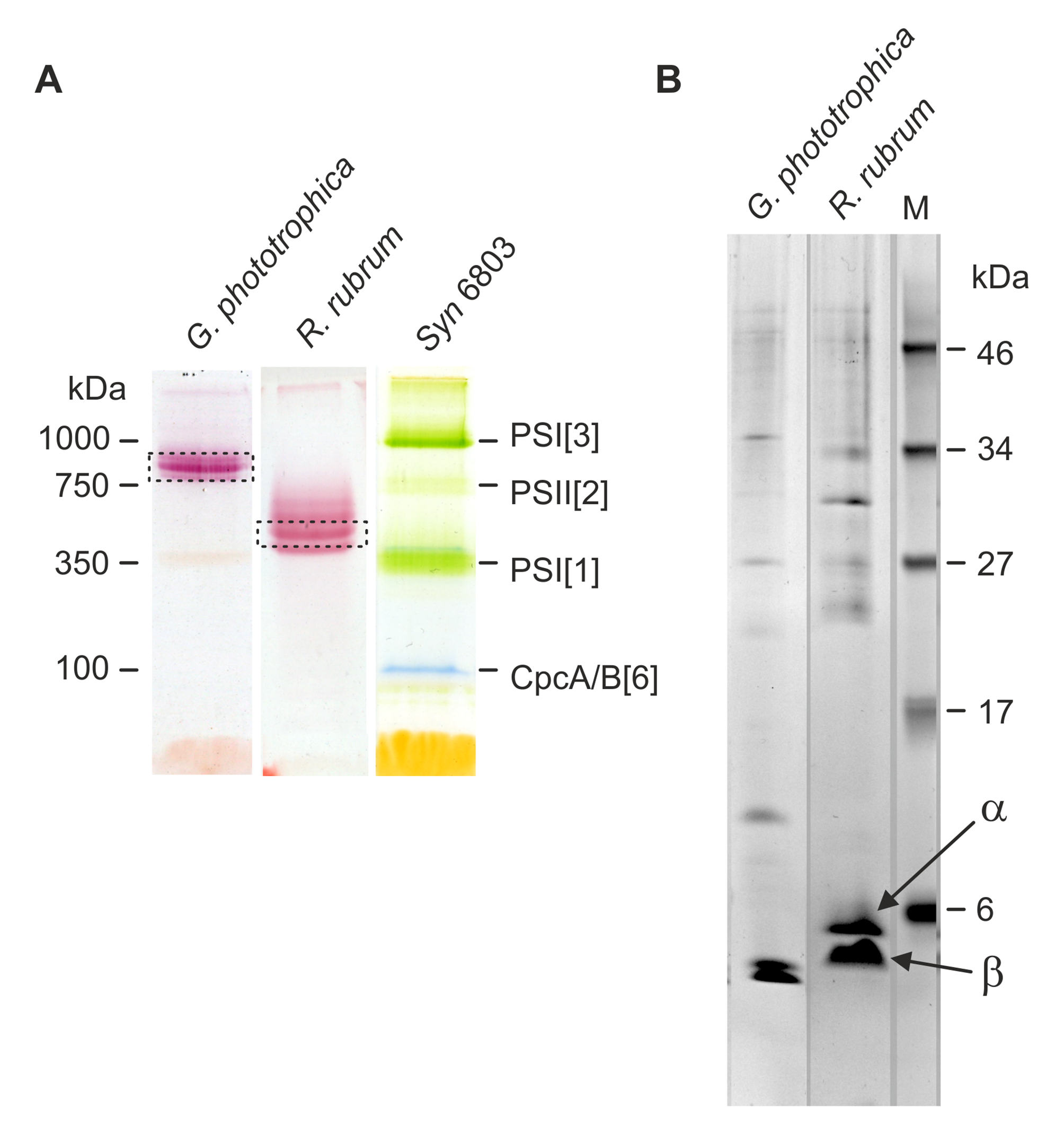

Supplement: S3 Fig — (A) Clear-native gel electrophoresis of G. phototrophica membrane complexes in comparison with membrane complexes of R. rubrum and Synechocystis sp. PCC 6803; the membranes were solubilized using 2% dodecyl-β-maltoside and loaded on 4%–14% clear-native gel (41). Abbreviations used: PSI[1] and PSI[3], monomer and trimer of PSI, respectively; PSII[2], dimer of PSII; CpcA/B[6], approximately 100 kDa heterohexamer of CpcA and CpcB phycobilinoproteins. (B) Colored bands corresponding to PS complexes of G. phototrophica and R. rubrum were cut from the native gel as indicated by dashed boxes in (A), incubated for 30 min in 2% SDS, and the proteins were separated by the gel electrophoresis. LH1 subunits of R. rubrum (α,β) are indicated by arrows. CpcA andCpcB, phycocyanine alpha and beta proteins; kDa, kiloDalton; PS, photosynthetic; SDS, sodium dodecyl sulfate. (TIF) [file pbio.2003943.s005.tif]
